# Extracorporeal membrane oxygenation as a bridge to lung transplantation: analysis of Korean organ transplantation registry (KOTRY) data

**DOI:** 10.1186/s12931-020-1289-2

**Published:** 2020-01-13

**Authors:** Ryoung-Eun Ko, Jin Gu Lee, Song Yee Kim, Young Tae Kim, Sun Mi Choi, Do Hyung Kim, Woo Hyun Cho, Seung-Il Park, Kyung-Wook Jo, Hong Kwan Kim, Hyo Chae Paik, Kyeongman Jeon, the Korean Organ Transplantation Registry Study Group

**Affiliations:** 1 Department of Critical Care Medicine, Samsung Medical Center, Sungkyunkwan University School of Medicine, Seoul, Korea; 2 Department of Thoracic and Cardiovascular Surgery, Severance Hospital, Yonsei University College of Medicine, Seoul, Korea; 3 Division of Pulmonary and Critical Care Medicine, Department of Internal Medicine, Severance Hospital, Yonsei University College of Medicine, Seoul, Korea; 4 Department of Thoracic and Cardiovascular Surgery, Seoul National University Hospital, Seoul National University College of Medicine, Seoul, Korea; 5 Division of Pulmonary and Critical Care Medicine, Department of Internal Medicine, Seoul National University Hospital, Seoul National University College of Medicine, Seoul, Korea; 6 Department of Thoracic and Cardiovascular Surgery, Pusan National University YangSan Hospital, Gyeongsangnam-do, Korea; 7 Department of Pulmonology and Critical Care Medicine, Pusan National University YangSan Hospital, Gyeongsangnam-do, Korea; 8 Department of Thoracic and Cardiovascular Surgery, Asan Medical Center, University of Ulsan College of Medicine, Seoul, Korea; 9 Department of Pulmonary and Critical Care Medicine, Asan Medical Center, University of Ulsan College of Medicine, Seoul, Korea; 10 Department of Thoracic and Cardiovascular Surgery, Samsung Medical Center, Sungkyunkwan University School of Medicine, Seoul, South Korea; 11 Division of Pulmonary and Critical Care Medicine, Department of Medicine, Samsung Medical Center, Sungkyunkwan University School of Medicine, 81 Irwon-ro, Gangnam-gu, Seoul, 06351 Korea

**Keywords:** Lung transplantation, Extracorporeal membrane oxygenations, Bridge to transplant, Treatment outcome

## Abstract

**Background:**

The use of extracorporeal membrane oxygenation (ECMO) as a bridge to lung transplantation has greatly increased. However, data regarding the clinical outcomes of this approach are lacking. The objective of this multicenter prospective observational cohort study was to evaluate lung transplantation outcomes in Korean Organ Transplantation Registry (KOTRY) patients for whom ECMO was used as a bridge to transplantation.

**Methods:**

Between March 2015 and December 2017, a total of 112 patients received lung transplantation and were registered in the KOTRY, which is a prospective, multicenter cohort registry. The entire cohort was divided into two groups: the control group (*n* = 85, 75.9%) and bridge-ECMO group (*n* = 27, 24.1%).

**Results:**

There were no significant differences in pre-transplant and intraoperative characteristics except for poorer oxygenation, more ventilator use, and longer operation time in the bridge-ECMO group. The prevalence of primary graft dysfunction at 0, 24, 48, and 72 h after transplantation did not differ between the two groups. Although postoperative hospital stays were longer in the bridge-ECMO group than in the control group, hospital mortality did not differ between the two groups (25.9% vs. 13.3%, *P* = 0.212). The majority of patients (70.4% of the bridge-ECMO group and 77.6% of the control group) were discharged directly to their homes. Finally, the use of ECMO as a bridge to lung transplantation did not significantly affect overall survival and graft function.

**Conclusions:**

Short- and long-term post-transplant outcomes of bridge-ECMO patients were comparable to recipients who did not receive ECMO.

## Background

Lung transplantation has become an accepted treatment for carefully selected patients with end-stage lung disease [1, 2]. However, due to a shortage of donors, the number of patients on waiting lists is growing rapidly and the average waiting time to lung transplantation has increased [3, 4]. Given these circumstances, there has been a corresponding increase in demand for mechanical ventilation (MV) and extracorporeal membrane oxygenation (ECMO) support to bridge these critically ill patients to lung transplantation.

MV is associated with risks of ventilator-associated pneumonia, ventilator-induced lung injury, and hemodynamic instability [5–8]. Furthermore, recent reports have indicated that waiting for lung transplantation with MV support is a risk factor for increased mortality compared without MV [9, 10]. Recently, due to advances in critical care management and improvements in technology regarding the safety profile and management of ECMO, the use of ECMO as a bridge to lung transplantation has steadily increased [11–13]. However, bridging to transplantation with ECMO has also been associated with major complications and increased in-hospital mortality [14, 15]. In addition, ECMO is invasive, requires anticoagulation for the duration of therapy, and can be associated with serious complications [16, 17]. Therefore, concerns remain about bridging patients with ECMO to lung transplantation. However, most data on lung transplantation after bridging with ECMO is drawn from retrospective, single-institution studies, and data describing long-term outcomes after lung transplantation are limited.

The objective of this study was to evaluate the mortality and long-term post transplantation outcomes of patients undergoing lung transplantation after bridging with ECMO by comparing them with non-bridge-ECMO patients through an analysis of Korean Organ Transplantation Registry (KOTRY) data.

## Methods

### Study design

KOTRY is a prospective, multicenter cohort registry that includes kidney, liver, pancreas, heart, and lung transplantations in Korea [18]. Lung transplanted patients from 5 institutions are enrolled in KOTRY. Patients are enrolled at the time of transplantation and then followed-up accordingly. Each participating institution inputs data through a web-based case report form according to a standardized protocol. Between March 2015 and December 2017, a total of 112 patients received lung transplantation and were registered in the KOTRY database. Written informed consent is obtained from each patient prior to transplantation. If patients are unable to provide consent due to disease severity, informed consent is obtained from a relative or legal representative. This KOTRY study was reviewed and approved by the Institutional Ethics Committees of each participating organization.

The clinical data of 112 patients received lung transplantation during study period were followed up until June 2018. The entire cohort was divided into two groups: the control group (*n* = 85) comprised recipients who did not require ECMO before lung transplantation and the bridge-ECMO group (*n* = 27) comprised recipients who were bridged to lung transplantation with ECMO. Post-transplant outcomes, including primary graft dysfunction (PGD) assessed and graded by the International Society for Heart and Lung Transplantation lung transplant injury grades [19], functional status at discharge, graft function, and survival up to 48 months after lung transplantation were assessed.

### Data collection and clinical outcomes

Information about transplant recipients, donors, transplant operations, and postoperative follow-up results were prospectively collected. Data for recipients including general demographic information, primary diagnosis, and pre-transplantation status, and data for donors including general demographic information, cause of brain death, and smoking status, were collected. Transplant surgery data including unilateral or bilateral lung transplantation, operation time, ischemic time, need for intraoperative hemodynamic support, and hemodynamic support type were collected. KOTRY also includes data about post-transplantation results including immediate complications, need for organ support, prevalence of primary graft dysfunction, serial pulmonary function, and outcomes such as the length of hospital stay, in-hospital and 6-month mortality, function status at discharge, and co-morbidities. The most recent information for each patient was collected at 3, 6, 9, and 12 months after discharge, and then annually. The follow-up data were collected from patients by the attending physician and stored using the web-based case report form.

### Statistical analysis

All data are presented as medians and interquartile ranges for continuous variables, and as numbers and percentages for categorical variables. We compared the clinical characteristics and outcomes of the two groups using the Mann-Whitney U test or Student’s t-test, as appropriate, for continuous variables and the chi-square test or Fisher’s exact test for categorical variables. Probability of survival curves for each group were estimated by the Kaplan-Meier method and compared by the log-rank test. Data were analyzed using IBM SPSS Statistics for Windows, version 23.0 (Armonk, NY, USA).

## Results

### Baseline characteristics

During the study period, a total of 112 patients underwent lung transplantation and were registered in KOTRY. The baseline characteristics of the patients are shown in Table 1. Among them, 71 (63.4%) were male and the median age of all patients was 58.0 (interquartile range, IQR 52.5–62.0) years. Idiopathic pulmonary fibrosis (53.6%) was the most common reason for lung transplantation, followed by connective tissue disease associated interstitial lung disease (17.9%) and bronchiolitis obliterans after hematopoietic stem cell transplantation (8.9%). One patient received simultaneous heart-lung transplantation due to Eisenmenger syndrome. All patients were receiving their first lung transplants. The pretransplant oxygenation with partial pressure of arterial oxygen (PaO_2_)/fraction of inspired oxygen (FiO_2_) ratio (PaO_2_/FiO_2_ ratio) was 224.0 (IQR 125.0–281.0). Thirty-nine patients (34.8%) received MV before lung transplantation and all patients in the bridge-ECMO group received MV simultaneously before lung transplantation. The median duration of bridging with ECMO was 11.0 (IQR 6.0–18.0) days in the bridge-ECMO group. Veno-venous mode (*n* = 24, 89%) was the most common type of ECMO used in ECMO-bridge group, followed by veno-venous-arterial in two and veno-arterial in one. All but four of cannulation configurations for veno-venous ECMO was femoro-femoral cannulation.

**Table 1:** Pre-transplant characteristics of lung transplant patients and donors

	Overall (*N* = 112)	Bridge-ECMO group (*n* = 27)	Non-bridge-ECMO group (*n* = 85)	*p*
Demographics				
Age, y	58.0 [52.5–62.0]	58.0 [53.0–62.0]	58.0 [52.0–62.0]	0.859
Sex (Male)	71 (63.4%)	21 (77.8%)	50 (58.8%)	0.121
BMI (kg/m^2^)	21.2 [19.0–23.5]	21.4 [18.7–23.4]	21.2 [19.1–23.5]	0.999
Primary diagnosis, n (%)				0.187
Idiopathic pulmonary fibrosis	60 (53.6%)	13 (48.1%)	47 (55.3%)	
Other fibrosis/Emphysema	7 (6.2%)	1 (3.7%)	6 (7.1%)	
CTD related interstitial lung disease	20 (17.9%)	5 (18.5%)	15 (17.6%)	
Bronchiolitis obliterans after HSCT	10 (8.9%)	2 (7.4%)	8 (9.4%)	
Acute respiratory distress syndrome	8 (7.1%)	5 (18.5%)	3 (3.5%)	
Others^a^	7 (6.2%)	1 (3.7%)	6 (7.1%)	
Pre-transplantation status				
PaO2/FiO2 ratio	224.0 [125.0–281.0]	110.0 [82.5–251.0]	229.0 [181.0–288.0]	0.008
LVEF, %	62.0 [59.0–67.0]	61.0 [56.0–65.0]	63.0 [59.0–68.0]	0.298
RVSP, mmHg	50.0 [41.0–66.0]	61.0 [47.0–88.0]	48.5 [40.5–64.5]	0.141
Preoperative MV, n (%)	39 (34.8%)	27 (100.0%)	12 (14.1%)	< 0.001
Wait-list duration (day)	69.5 [18.5–127.5]	27.0 [10.5–40.5]	87.0 [35.0–145.0]	< 0.001
Donor demographics				
Sex (Male)	66 (58.9%)	16 (59.3%)	50 (58.8%)	1.000
Age, y	41.5 [32.0–49.0]	38.0 [33.5–47.0]	43.0 [31.0–50.0]	0.464
BMI (kg/m^2^)	22.1 [20.1–24.2]	20.9 [20.0–23.2]	22.7 [20.1–25.1]	0.122
Donor cause of brain death, n (%)				0.053
Trauma	37 (33.0%)	12 (44.4%)	25 (29.4%)	
Underlying disease progression	27 (24.1%)	8 (29.6%)	19 (22.4%)	
Suicide	25 (22.3%)	7 (25.9%)	18 (21.2%)	
Donor current smoker, n (%)	46 (41.1%)	7 (25.9%)	39 (45.9%)	0.043

*BMI* body mass index, *CTD* connective tissue disease, *HSCT* hematopoietic stem cell transplantation, *FEV1* forced expiratory volume in 1 s, *PaO2* arterial oxygen tension, *FiO2* fractional inspired oxygen, *LVEF* left ventricular ejection fraction, *RVSP* right ventricular systolic pressure, *MV* mechanical ventilation, *ECMO* extracorporeal membrane oxygenation

^a^Others include 3 bronchiectasis patients, 2 lymphangioleiomyomatoses patients, one Eisenmenger syndrome patient who received both heart and lung transplants, and one secondary pulmonary arterial hypertension patient

The median age of donors was 41.5 (IQR 32.0–49.0) years and the most common cause of brain death was trauma (33.0%). Forty-six (41.1%) donors were current smokers. All organs were from deceased donors.

### Intra-operation characteristics

The intra-operation characteristics of the enrolled patients are shown in Table 2. One hundred and eight (96.4%) patients underwent bilateral lung transplantation. Total ischemic time for the right lung was 232.0 (IQR 180.0–338.5) minutes and total ischemic time for the left lung was 305.0 (IQR 258.0–372.0) minutes. The median operation time of all patients was 480.0 (IQR 378.0–612.5) minutes. The median operation time in the bridge-ECMO group (575.0 min, IQR 474.0–690.0) was longer than in the control group (455.0 min, IQR 364.0–555.0) (*P* <  0.001). Seventy-nine (70.5%) patients required mechanical support with ECMO during the operation.

**Table 2:** Intra-operative characteristics

	Overall (N = 112)	Bridge-ECMO group (*n* = 27)	Non-bridge-ECMO group (*n* = 85)	*p*
Bilateral lung transplantation, n (%)	108 (96.4%)	26 (96.3%)	82 (96.5%)	1.000
Operation Time, min	480.0 [378.0–612.5]	575.0 [474.0–690.0]	455.0 [364.0–555.0]	< 0.001
Total ischemic time, right, min	232.0 [180.0–338.5]	280.0 [230.5–363.0]	223.0 [172.0–311.0]	0.008
Total ischemic time, left, min	305.0 [258.0–372.0]	331.0 [250.0–372.0]	300.0 [259.0–372.0]	0.849
Intraoperative CPB support, n (%)	36 (32.1%)	12 (44.4%)	24 (28.2%)	0.182
Intraoperative ECMO support, n (%)	79 (70.5%)	20 (74.1%)	59 (69.4%)	0.825
ECMO type, n (%)				< 0.001
Veno-venous	11 (13.9%)	9 (45.0%)	2 (3.4%)	
Veno-arterial	68 (86.1%)	11 (55.0%)	57 (96.6%)	

*ECMO* extracorporeal membrane oxygenation, *CPB* cardiopulmonary bypass

### Post-transplantation outcomes

After lung transplantation, immediate complications developed in 48 (42.9%) patients (Table 3). The most common immediate complication was infection (35.4%), followed by post operation bleeding (33.3%). The frequency of immediate complications was not different between the bridge-ECMO group and non-bridge-ECMO group. However, postoperative length of stay in the intensive care unit was longer in the bridge-ECMO group (33.0 days, IQR 23.0–43.5) compared with the non-bridge-ECMO group (9.0 days, IQR 6.0–16.0) (*P* <  0.001). In addition, the time from transplantation to hospital discharge was longer in the bridge-ECMO group (46.0 days, IQR 38.5–68.5) compared with the non-bridge-ECMO group (35.0 days, IQR 25.0–67.0) (*P* = 0.030). The hospital mortality for all patients was 16.4%, with no significant difference between the two groups (25.9% in bridge-ECMO group and 13.3% in non-bridge-ECMO group, *P* = 0.212). Partially dependent (43.8%) was the most common functional status at discharge, followed by fully independent (33.9%), and was not different between the two groups. The majority of patients (70.4% of bridge-ECMO group and 77.6% of non-bridge-ECMO group) were discharged to their homes. Only 5 patients (4.5%) required tracheostomy at discharge.

**Table 3:** Post-transplantation outcomes

	Overall (N = 112)	Bridge-ECMO group (n = 27)	Non-bridge-ECMO group (n = 85)	*p*
Immediate complications^a^, n (%)	48 (42.9%)	15 (55.6%)	33 (38.8%)	0.191
Post operation bleeding	16 (33.3%)	7 (46.7%)	9 (27.3%)	0.322
Reoperation	14	6	8	
Conservative management	2	1	1	
Infection	17 (35.4%)	4 (26.7%)	13 (39.4%)	0.597
Airway complication	4 (8.3%)	2 (13.3%)	2 (6.1%)	0.778
Total ICU length of stay, day	13.0 [6.0–31.0]	33.0 [23.0–43.5]	9.0 [6.0–16.0]	< 0.001
Time interval between transplantation and discharge, day	39.0 [26.5–67.0]	46.0 [38.5–68.5]	35.0 [25.0–67.0]	0.030
Hospital mortality, n (%)	18 (16.4%)	7 (25.9%)	11 (13.3%)	0.212
Function status at discharge, n (%)				0.295
Fully independent	38 (33.9%)	10 (37.0%)	28 (32.9%)	
Partially dependent	49 (43.8%)	8 (29.6%)	41 (48.2%)	
Fully dependent	5 (4.5%)	2 (7.4%)	3 (3.5%)	
Tracheostomy at discharge, n (%)	5 (4.5%)	1 (3.7%)	4 (4.7%)	0.703
Final destination, n (%)				0.405
Home	85 (75.9%)	19 (70.4%)	66 (77.6%)	
Other hospital	7 (6.2%)	1 (3.7%)	6 (7.1%)	
Developed co-morbidity^b^, n (%)				
Diabetes mellitus	24 (27.0%)	5 (27.8%)	19 (26.8%)	1.000
Hypertension	16 (18.0%)	3 (16.7%)	13 (18.3%)	1.000
PTLD	1 (1.1%)	0 (0.0%)	1 (1.4%)	1.000
Cancer	1 (1.1%)	0 (0.0%)	1 (1.4%)	1.000
CKD with hemodialysis	3 (2.7%)	0 (0.0%)	3 (3.5%)	0.433
6 months mortality, n (%)	27 (24.1%)	9 (33.3%)	18 (21.2%)	0.304

*ECMO* extracorporeal membrane oxygenation, *RRT* renal replacement therapy, *ICU* intensive care unit, *PTLD* post-transplant lymphoproliferative disease

^a^Immediate complication evaluated in ICU complication after lung transplantation

^b^Co-morbidity evaluated at 6 months after lung transplantation

As shown in Table 4, the prevalence of PGD was not significantly different between the bridge-ECMO group and non-bridge-ECMO group at 0 h, 24 h, 48 h, and 72 h after lung transplantation (*P* = 0.255, *P* = 0.481, *P* = 0.817, and *P* = 0.561 respectively).

**Table 4:** Prevalence of primary graft dysfunction after lung transplantation

	Overall (N = 112)	Bridge-ECMO group (n = 27)	Non-bridge-ECMO group (n = 85)	*p*
0 h				0.255
PGD 0	89 (79.5%)	25 (92.6%)	64 (75.3%)	
PGD 1	1 (0.9%)	0 (0.0%)	1 (1.2%)	
PGD 2	6 (5.4%)	1 (3.7%)	5 (5.9%)	
PGD 3	16 (14.3%)	1 (3.7%)	15 (17.6%)	
24 h				0.481
PGD 0	96 (85.7%)	25 (92.6%)	71 (83.5%)	
PGD 1	5 (4.5%)	1 (3.7%)	4 (4.7%)	
PGD 2	4 (3.6%)	1 (3.7%)	3 (3.5%)	
PGD 3	7 (6.2%)	0 (0.0%)	7 (8.2%)	
48 h				0.817
PGD 0	99 (88.4%)	25 (92.6%)	74 (87.1%)	
PGD 1	5 (4.5%)	1 (3.7%)	4 (4.7%)	
PGD 2	6 (5.4%)	1 (3.7%)	5 (5.9%)	
PGD 3	2 (1.8%)	0 (0.0%)	2 (2.4%)	
72 h				0.561
PGD 0	99 (88.4%)	25 (92.6%)	74 (87.1%)	
PGD 1	4 (3.6%)	0 (0.0%)	4 (4.7%)	
PGD 2	7 (6.2%)	2 (7.4%)	5 (5.9%)	
PGD 3	2 (1.8%)	0 (0.0%)	2 (2.4%)	

*ECMO* extracorporeal membrane oxygenation, *PGD* primary graft dysfunction

**Fig. 1:**
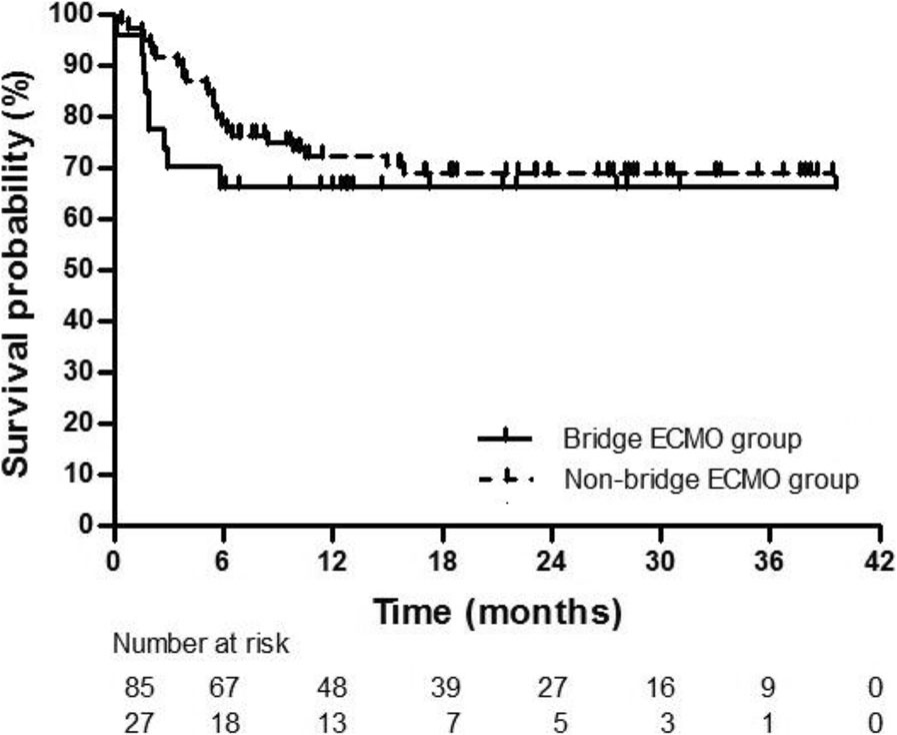
Kaplan-Meier analysis of survival after lung transplantation

**Fig. 2:**
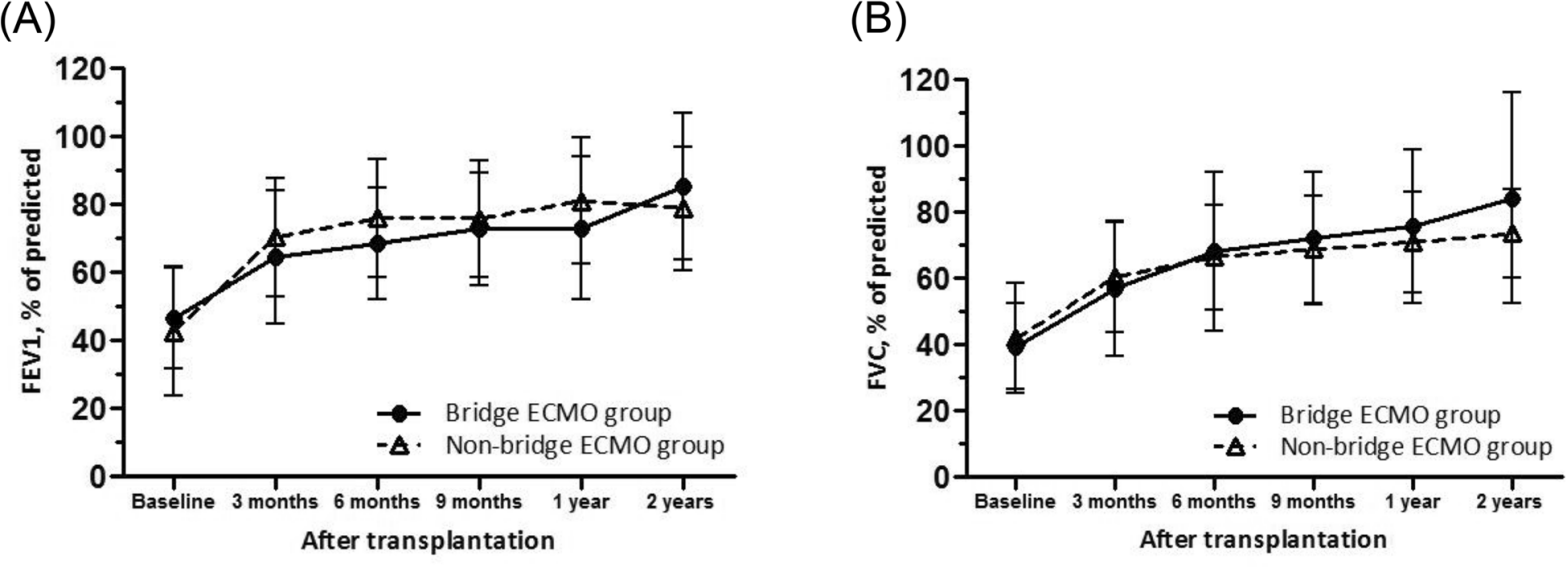
Forced expiratory volume in 1 s (FEV_1_) (A) and forced vital capacity (FVC) (B), expressed as percent of predicted, after lung transplantation. Error bars depict the interquartile range of the median

Survival rate at 6 months after lung transplantation was 75.9%, and was not significantly different between the bridge-ECMO group (66.6%) and non-bridge-ECMO group (78.8%) (*P* = 0.304). Bridging with ECMO prior to lung transplantation did not significantly affect overall survival (Fig. 1). Although the probability of survival for the bridge-ECMO group appeared to decrease in the first few months post-transplantation, this difference was not statistically significant (*P* = 0.139, log-rank test). In addition, there were no significant differences in post-transplant lung function between the two groups at 3 months, 6 months, 9 months, 12 months, or 24 months postoperatively (Fig. 2).

## Discussion

In this multicenter prospective observational study, we found that there were no significant differences in immediate postoperative complications, development and severity of PGD, functional status at discharge, long-term survival, or lung function in patients who received bridging with ECMO compared with the control group, despite longer operation time, longer ICU stay, and longer hospitalization after lung transplantation in the former group.

ECMO support improves outcomes in patients with life-threatening respiratory failure [11] and the application of ECMO as a rescue therapy is expanding in clinical practice [20]. In addition, ECMO has become a lifesaving intervention for a subset of rapidly deteriorating patients with end-stage lung disease, which offers optimizing gas exchange and end-organ perfusion to patients who might otherwise die before a suitable donor lung becomes available. Although bridging with ECMO is generally associated with a greater perioperative risk and poorer long-term survival [21], ECMO allows actively deteriorating and severely ill patients with end-stage lung disease to remain eligible for lung transplantation.

During the last decade, several studies aimed to evaluate survival outcomes between bridge-ECMO patients and non-bridge-ECMO patients to demonstrate the efficacy of using ECMO as a bridge strategy. Toyoda et al. detected no survival difference between the bridge-ECMO group (*n* = 24) and non-bridge-ECMO group (*n* = 691) of 74% versus 83%, respectively, at 1 year after transplantation [22]. Schechter et al. showed that 1-year survival was not different between bridge only with ECMO patients and control patients (70.4% versus 84.2%) [23]. In contrast, Inci et al. showed worse overall and 3 month conditional survivals in the bridge-ECMO group (*n* = 26) versus the non-bridge-ECMO group (*n* = 160) (68% versus 85%, *p* = 0.001; 86% versus 92%, *p* = 0.03, respectively) [15]. In the present study, the 6-month mortality of all patients was 24.1% and there was no significant difference between the bridge-ECMO group and non-bridge-ECMO group in this respect (33.3 and 21.2%, *P* = 0.304). The strength of our study is that recent, multi-institutional data for lung transplantation and a large sample of bridge-ECMO patients were included. These results indicate that bridging with ECMO is effective for patients awaiting lung transplantation due to the recent evolution of ICU care and ECMO management.

In this study, we also provide valuable information about short-term post transplantation outcomes. Despite technical improvements, ECMO is associated with risks of complications including hemolysis and need for transfusion, cardiovascular dysfunction, bleeding due to anticoagulation, and thrombosis formation. Furthermore, ICU admission for ECMO management leads to ICU-acquired weakness and infection associated with catheter or ICU care [24, 25]. Our results indicate that post-operative bleeding (46.7%) is the most common immediate complication in bridge-ECMO patients, while infection (39.4%) is the most common in the non-bridge-ECMO group, although there were no significant differences in number of immediate complications after transplantation including post-operative bleeding, infection, and airway complications between the two groups. All patients but two showed feasible functional status at discharge and all patients but one were discharged home in the bridge-ECMO group. These results indicate that bridging with ECMO is feasible for patients awaiting lung transplantation by considering not only survival but also quality of life after discharge.

In the present study, the long-term outcomes of lung transplantation after bridging with ECMO were considered acceptable. KOTRY collects data for each patient serially at 3, 6, 9, 12 months after discharge, and then annually. However, our analysis includes only 2 years of follow-up data since the inclusion of lung transplant patients in KOTRY was initiated only in 2015. Pulmonary function, including predicted FEV1 and FVC, showed no significant differences between the bridge-ECMO group and non-bridge-ECMO group at 3 months, 6 months, 9 months, 12 months, or 24 months follow-up. Co-morbidities including hypertension, diabetes, and maintenance hemodialysis that developed within 2 years after lung transplantation did not significantly differ between the two groups. These findings suggest that long-term prognosis for lung transplant patients after bridge ECMO is acceptable, if lung transplantation is successful.

Although the results of this study provide additional information on short- and long-term outcomes of lung transplantation after bridging with ECMO in a relatively large sample from a prospective multicenter registry, the study has several limitations that should be acknowledged. First, because of the observational nature of the study, our findings remain prone to various biases. We used a national multicenter designed to improve the generalizability of our findings, but there is a potential risk of selection bias. In addition, differences in pre-transplantation status between the two groups should be considered, which might influence the clinical outcomes. Although the bridge-ECMO group was more severely ill before transplantation, however, transplantation outcomes was comparable. Second, because KOTRY was designed to collect the follow up data of lung transplantation, detailed information on the clinical status prior to ECMO, ECMO management including case selection, and rehabilitation prior to transplantation was not systematically collected. Third, KOTRY includes a large number of bridge-ECMO patients compared to other studies [26–28]. The high rate of bridge with ECMO in our study may reflect the Korean lung allocation system based firstly on urgency of transplant [28], which is different from the European lung allocation score system based on the expected benefit after lung transplantation as well as the disease severity. Under a medical urgency-based allocation system regardless of post-transplant survival in Korea, therefore, patients with ECMO on waiting list are given the highest priority for transplantation. However, this is what allowed a large number of lung transplant patients who underwent bridging with ECMO to be enrolled. Fourth, KOTRY is not the only source of data regarding lung transplantation in Korea. Compared with the Korean Network for Organ Sharing data, only 49% of all patients who received lung transplantation in Korea during the study period were registered in the KOTRY. Finally, KOTRY enrolls patients at the time of transplantation, and we were unable to analyze patients who died while waiting for lung transplantation while on ECMO support.

## Conclusion

In conclusion, lung transplantation after bridging with ECMO leads to acceptable patient outcomes. However, current evidence does not permit firm conclusions regarding the efficacy of bridging with ECMO and further systematic multicenter trials among carefully selected patients with end-stage lung disease are needed.

## Data Availability

The data that support the findings of this study are available on request from the corresponding author. The data are not publicly available due to privacy or ethical restrictions.

## Abbreviations

ECMO: Extracorporeal membrane oxygenation
FEV1: Forced expiratory volume in 1 s
FVC: Forced vital capacity
ICU: Intensive care unit
KOTRY: Korean organ transplantation registry
PGD: Primary graft dysfunction

## References

[1] Kotloff RM, Thabut G (2011). Lung transplantation. Am J Respir Crit Care Med.

[2] Arcasoy SM, Kotloff RM (1999). Lung transplantation. N Engl J Med.

[3] Yeo HJ, Yoon SH, Lee SE, Jeon D, Kim YS, Cho WH (2017). Current status and future of lung donation in Korea. J Korean Med Sci.

[4] Klein AS, Messersmith EE, Ratner LE, Kochik R, Baliga PK, Ojo AO (2010). Organ donation and utilization in the United States, 1999-2008. Am J Transplant.

[5] Hoeper MM, Granton J (2011). Intensive care unit management of patients with severe pulmonary hypertension and right heart failure. Am J Respir Crit Care Med.

[6] Kalanuria AA, Ziai W, Mirski M (2014). Ventilator-associated pneumonia in the ICU. Crit Care.

[7] Katira BH, Giesinger RE, Engelberts D, Zabini D, Kornecki A, Otulakowski G (2017). Adverse heart-lung interactions in ventilator-induced lung injury. Am J Respir Crit Care Med.

[8] Slutsky AS, Ranieri VM (2013). Ventilator-induced lung injury. N Engl J Med.

[9] Mason DP, Thuita L, Nowicki ER, Murthy SC, Pettersson GB, Blackstone EH (2010). Should lung transplantation be performed for patients on mechanical respiratory support? The US experience. J Thorac Cardiovasc Surg.

[10] Singer JP, Blanc PD, Hoopes C, Golden JA, Koff JL, Leard LE (2011). The impact of pretransplant mechanical ventilation on short- and long-term survival after lung transplantation. Am J Transplant.

[11] Peek GJ, Mugford M, Tiruvoipati R, Wilson A, Allen E, Thalanany MM (2009). Efficacy and economic assessment of conventional ventilatory support versus extracorporeal membrane oxygenation for severe adult respiratory failure (CESAR): a multicentre randomised controlled trial. Lancet..

[12] Javidfar J, Bacchetta M (2012). Bridge to lung transplantation with extracorporeal membrane oxygenation support. Curr Opin Organ Transplant.

[13] Tipograf Yuliya, Salna Michael, Minko Elizaveta, Grogan Eric L., Agerstrand Cara, Sonett Joshua, Brodie Daniel, Bacchetta Matthew (2019). Outcomes of Extracorporeal Membrane Oxygenation as a Bridge to Lung Transplantation. The Annals of Thoracic Surgery.

[14] Hayanga JW, Aboagye JK, Hayanga HK, Luketich JD, D'Cunha J (2016). Extracorporeal membrane oxygenation as a bridge to lung re-transplantation: is there a role?. J Heart Lung Transplant.

[15] Inci I, Klinzing S, Schneiter D, Schuepbach RA, Kestenholz P, Hillinger S (2015). Outcome of extracorporeal membrane oxygenation as a bridge to lung transplantation: an institutional experience and literature review. Transplantation..

[16] Makdisi G, Wang IW (2015). Extra corporeal membrane oxygenation (ECMO) review of a lifesaving technology. J Thorac Dis..

[17] Chiumello D, Coppola S, Froio S, Colombo A, Del Sorbo L (2015). Extracorporeal life support as bridge to lung transplantation: a systematic review. Crit Care.

[18] Lee JG, Kim SY, Kim YT, Lee HJ, Park S, Choi SM (2018). First report of the Korean lung transplantation registry. Transplant Proc.

[19] Christie JD, Carby M, Bag R, Corris P, Hertz M, Weill D (2005). Report of the ISHLT working group on primary lung graft dysfunction part II: definition. A consensus statement of the International Society for Heart and Lung Transplantation. J Heart Lung Transplant.

[20] Thiagarajan RR, Barbaro RP, Rycus PT, McMullan DM, Conrad SA, Fortenberry JD (2017). Extracorporeal life support organization registry international report 2016. ASAIO J.

[21] Gupta P, McDonald R, Chipman CW, Stroud M, Gossett JM, Imamura M (2012). 20-year experience of prolonged extracorporeal membrane oxygenation in critically ill children with cardiac or pulmonary failure. Ann Thorac Surg.

[22] Toyoda Y, Bhama JK, Shigemura N, Zaldonis D, Pilewski J, Crespo M (2013). Efficacy of extracorporeal membrane oxygenation as a bridge to lung transplantation. J Thorac Cardiovasc Surg.

[23] Schechter MA, Ganapathi AM, Englum BR, Speicher PJ, Daneshmand MA, Davis RD (2016). Spontaneously breathing extracorporeal membrane oxygenation support provides the optimal bridge to lung transplantation. Transplantation..

[24] van Vught LA, Klein Klouwenberg PM, Spitoni C, Scicluna BP, Wiewel MA, Horn J (2016). Incidence, risk factors, and attributable mortality of secondary infections in the intensive care unit after admission for sepsis. JAMA..

[25] Kress JP, Hall JB (2014). ICU-acquired weakness and recovery from critical illness. N Engl J Med.

[26] Hayanga AJ, Aboagye J, Esper S, Shigemura N, Bermudez CA, D'Cunha J (2015). Extracorporeal membrane oxygenation as a bridge to lung transplantation in the United States: an evolving strategy in the management of rapidly advancing pulmonary disease. J Thorac Cardiovasc Surg.

[27] Gulack BC, Hirji SA, Hartwig MG (2014). Bridge to lung transplantation and rescue post-transplant: the expanding role of extracorporeal membrane oxygenation. J Thorac Dis.

[28] Yu WS, Kim SY, Kim YT, Lee HJ, Park S, Choi SM (2019). Characteristics of lung allocation and outcomes of lung transplant according to the Korean urgency status. Yonsei Med J.

